# Lived Experience of Parents with Technology-Dependent Children: A Phenomenological Study

**DOI:** 10.1177/23333936241288858

**Published:** 2024-11-15

**Authors:** Sara Raquel Machado Lemos, Luísa Maria Costa Andrade, Lígia Maria Monteiro Lima, Isabel Maria Fernández-Medina, Maria Do Céu Aguiar Barbieri-Figueiredo

**Affiliations:** 1 University of Porto, Porto, Portugal; 2 Center for Health Technology and Services Research (CINTESIS), Porto, Portugal; 3 Nursing School of Porto, Porto, Portugal; 4 Center for Health Technology and Services Research (CINTESIS@RISE), Porto, Portugal; 5 University of Almeria, Almeria, Andalucia, Spain; 6 University of Huelva, Huelva, Spain

**Keywords:** lived experience, qualitative research, chronic illness, parents, technology dependent children, Portugal

## Abstract

Caring for technology-dependent children significantly impacts the family unit, a topic that has not been researched in Portugal. Understanding this impact can help nurses to improve their interventions. This study aimed to describe the lived experiences of parents with technology-dependent children using a qualitative descriptive design. We applied Giorgi’s approach to analyze 10 individual semi-structured interviews with parents recruited through a children’s hospital in Portugal. Our analysis identified two major themes: (1) Discovering a new parenthood: parent and caregiver, comprising two subthemes: Family reorganization and Learning to use a medical device; (2) Reconciling daily life with the needs of the technology-dependent children, comprising two subthemes: Importance of support systems and Experiencing difficulties. These results indicate that parents experience a wide range of concerns and challenges in managing medical devices, starting from the moment the need for a device is communicated and continuing through the process of learning and providing care. This journey involves significant changes in family dynamics and is marked by sacrifice and adaptation, supported by family, formal support systems, and healthcare professionals. Our findings provide valuable insights into the vulnerabilities faced by these parents and highlight how nursing care can enhance the quality of care for these families.

## Background

Healthcare delivery to high-risk newborns and children with chronic conditions has advanced significantly in recent decades, contributing to increased child survival rates but also to a higher incidence of childhood disabilities and dependence on medical devices (Barone et al., 2020; Forjaz de Lacerda & Gomes, 2017). These children require complex care involving technologies such as mechanical ventilation, renal dialysis, medication via central catheter, respiratory support, nutritional support, or other medical devices that support bodily functions and can be used alone or in combination (Spratling, 2015). Technology-dependent or medically fragile children can have a wide variety of chronic conditions (Toly, Blanchette, & Musil, 2019). In the United States, it is estimated that there are over 600,000 technology-dependent children (U.S. Department of Health and Human Services, Health Resources and Services Administration, Maternal and Child Health Bureau, 2013). Given the advances in medical technology and healthcare, it is likely that this population has increased significantly since then (Mitchell et al., 2022). In Portugal, the latest evidence shows that technological support is present in 26% of hospitalized children with chronic conditions (Romana et al., 2023).

The care of technology-dependent children is multifaceted and requires highly variable and complex care from healthcare professionals and family members (Barone et al., 2020; Curran et al., 2020). These caregiving demands can contribute to family overload and lead to physical, emotional, and financial strain, with a negative impact on the family’s quality of life and well-being (Caicedo, 2015; Chan et al., 2019; González et al., 2017). Previous research has found that families report challenges such as lack of time (Amar-Dolan et al., 2020; Choi et al., 2020), social isolation (Amar-Dolan et al., 2020; González et al., 2017), lack of personal and professional growth (Choi et al., 2020; González et al., 2017), financial issues (Choi et al., 2020; González et al., 2017), anxiety, depression, emotional stress (Choi et al., 2020; González et al., 2017; Nishigaki et al., 2016; Toly et al., 2022), sleep deprivation (Choi et al., 2020), and family dysfunction (González et al., 2017).

Despite these challenges, these families tend to be highly resilient when facing complex life events, particularly when supported by tailored healthcare services and quality care from healthcare professionals (Toledano-Toledano et al., 2020; Yang et al., 2022). The Portuguese health system provides universal, tax-funded healthcare that aims to guarantee equal access to healthcare services for all citizens. It is also a system that strongly focuses on maternal and child health (OECD/European Observatory on Health Systems and Policies, 2021). Although Portugal offers comprehensive services to support the health and well-being of families with technology-dependent children, there is a lack of comprehensive research to understand the specific needs and challenges these families face.

Understanding the lived experiences of families with technology-dependent children is essential for improving nursing care and supporting these families (Breneol et al., 2019). While previous studies have explored these experiences in other countries, including the United States (Toly, Blanchette, & Musil, 2019) and the United Kingdom, whose system shares many similarities with Portugal (Mitchell et al., 2022), a gap remains in understanding the specific context of Portuguese families. This study aims to fill this gap, providing knowledge that might improve nursing interventions and support care tailored to the needs of these families in Portugal. Thus, this study aims to describe the lived experiences of parents with technology-dependent children.

## Methods

### Design

This study used a qualitative descriptive phenomenological design conducted using Giorgi’s et al. (2017) approach. The phenomenology approach seeks to describe the essence of a phenomenon as lived by a person who had the experience and to comprehend the meaning of this experience from the participants’ perspectives (Giorgi et al., 2017).

### Participants and Setting

The study was conducted in an outpatient department of a university hospital in Northern Portugal that provided specialized care for treating chronic conditions, particularly for technology-dependent children. For eligibility criteria, see Table 1.

**Table 1.** Inclusion and Exclusion Criteria.

Inclusion criteria	Exclusion criteria
Parent or family carer aged ≥ 18 years	Parent or family carer aged < 18 years
The child has one or more diagnoses of a chronic condition	The child has a diagnosis of oncologic conditions and/or is in palliative care
The child is dependent on medical device	The child is not dependent on medical device
Family member has experience living with technology-dependent children at home without time limits	Family member has no experience of living with technology-dependent children at home

A purposive sampling approach was used to recruit participants who met the inclusion criteria. The study’s information was disseminated to participants by the author (S.L.) and the nursing staff from the selected service. Interested parents contacted the principal researcher via email or phone and were screened for eligibility. Following informed consent, a meeting was scheduled to conduct an interview.

### Data Collection

Data collection was carried out between October 2022 and May 2023 using semi-structured interviews. All the interviews were conducted by the study’s first author (S.L.), who had previous experience and training in pediatric nursing and qualitative research. The interviews were conducted using a script with open-ended questions based on the reviewed literature and the researchers’ previous clinical experience (Table 2).

**Table 2.** Interview Protocol.

Stage of the interview	Topic	Content/example questions
Introduction	Objective	To understand the experience of families caring for technology-dependent children.
	Ethical aspects	Provide information about voluntary participation, confidentiality, anonymity, possibility of withdrawal or not answering, and recording consent.
Start	Opening questions	Could you please tell me about your experience of caring for your child? What kind of care does your child need on a daily basis?
Development	Specific questions	From the time of the diagnosis up to today, how have you adapted to caring for your technology-dependent child? How has the adaptation been for all the family members involved in the caregiving process?
Closing	Final question	Is there anything you would like to add about your experience while caring for your child? Is there anything that I did not address and you would like to add?
	Acknowledgments	We would like to thank you for your time. Your account will be incredibly useful to us.

Interviews were carried out with the participants (*n* = 10), with eight taking place in a private room in the hospital (*n* = 8) and two in the participants’ homes (*n* = 2), depending on the family’s preferences. The interviews had an average duration of 1 hr, and each participant was interviewed only once. All interviews were audio-recorded and later transcribed verbatim by the same team member who conducted the interviews (S.L.) and was involved in the data analysis, ensuring a thorough understanding of the content until data saturation was reached. Data saturation was reached with participant number eight; however, two more interviews were conducted to confirm that no new themes emerged. Using Giorgi’s phenomenological approach, the analysis focused on identifying the essential structures of the phenomenon. As highlighted by Norlyk and Harder (2010), we emphasized the essence of the lived experiences, ensuring that the findings captured the deeper meanings inherent in the participants’ narratives.

### Data Analysis

Data were analyzed following Giorgi’s (2017) five-step approach using the analysis software ATLAS.it 23.0. The analysis was conducted by the first author (S.L.) and the second author (L.A.). In step 1, the transcripts were read several times to gain a deep understanding of participants’ experiences, forming an overall impression of the phenomenon under study. In step 2, units of meaning were identified within the transcripts. These meaning units are significant segments of text that capture essential aspects of participants’ experiences. In step 3, these units of meaning re-examined and then transformed from the participants’ natural expressions into “phenomenological expressions,” capturing the essence of their experiences. In step 4, the transformed meaning units were synthesized to identify common meanings, check their interdependencies, and organize the phenomenological expressions into a cohesive description that reflected the overall structure of the participants’ experiences. Finally, in step 5, the authors (S.L., L.A., L.L. and M.C.F.-B.) comprehensively identified the essential characteristics of the phenomenon by transforming common language into phenomenological language through themes and subthemes. The results were then integrated into a descriptive statement that encapsulated the lived experiences of families with technology-dependent children. Following Giorgi’s rigorous phenomenological approach, we systematically explored and described the lived experiences of families with technology-dependent children and drew meaningful insights from our analysis.

### Rigor

The rigor of the study was ensured by using a combination of the criteria proposed by Guba and Lincoln (2005) with those specifically aligned with phenomenological research, as highlighted by Giorgi’s approach (Norlyk & Harder, 2010). Credibility was achieved through developing a trusting relationship with the participants, which encouraged open sharing experiences. In addition, experts on the qualitative research team (L.A.; L.L.; I. F.-M. and M.C.F.-B.) evaluated and directed the research process, ensuring that the descriptions aligned with the interview data. To support confirmability, the first author (S.L.), who had no prior relationship with the participants, conducted all interviews using a consistent guide, and multiple researchers were engaged in the analysis of data. Data analysis followed Giorgi’s method, focusing on transforming participants’ descriptions into phenomenological expressions to capture the essence of their experiences. Any differences in interpretation were discussed and resolved by two researchers (S.L. and L.A.), ensuring dependability. Finally, a detailed description of the context, participants, and method was provided to assist transferability. The Consolidated Criteria for Reporting Qualitative Research (COREQ) checklist (Tong et al., 2007) was used to guide the reporting of this research.

### Ethical Considerations

This study was approved by the Ethics Committee of the University Hospital of Northern Portugal (No. 18-22). The research was carried out respecting the ethical principles for medical research, including human subjects, established in the Declaration of Helsinki. All participants were informed of the study’s aim and signed an informed consent prior to the interview. The participants’ anonymity, privacy, and confidentiality were guaranteed.

## Findings

The final sample consisted of ten parents (nine mothers and one father) aged between 31 and 57 years old (*M* = 39.6; *SD* = 7.34). All parents were married. Most parents had secondary education (*n* = 8). Families had between one and four children. Half of the parents were employed, and the other half cared for their children full-time. In relation to the children’s age, the majority were between 3 and 6 years old (*n* = 3) or 14 and 18 years old (*n* = 3). Children had one or more than one type of medical device, the most frequent of which was the Central Venous Catheter (CVC; *n* = 8). Further details about the demographic characteristics of the parents and their children are presented in Table 3.

**Table 3.** Sociodemographic Characteristics of the Parents and Their Children.

No. participant	Parents’ sex	Parents’ occupation	Parents’ level of education	Child’s sex	Child’s age (years)	Child’s diagnosis	Child’s type of medical device
P1	Female	Employee	Secondary school	Female	4	Hurler syndrome	CVC Hearing aids
P2	Female	Employee	Secondary school	Female	7	Hurler syndrome	CVC
P3	Female	Housewife	Secondary school	Female	1	Atypical hemolytic uremic syndrome Epilepsy	CVC
P4	Male	Employee	Secondary school	Female	6	Pompe disease	CVC PEG BiPAP
P5	Female	Housewife	Secondary school	Male	3	Short bowel syndrome Cerebral palsy Epilepsy	CVC
P6	Female	Housewife	University degree	Female	1	Short bowel syndrome	CVC
P7	Female	Housewife	Secondary school	Male	12	Ultrashort bowel syndrome	CVC G-button Colostomy Abdominal drainage
P8	Female	Employee	Secondary school	Female	16	Diabetic type I	Insulin pump therapy
P9	Female	Housewife	Secondary school	Female	16	Rett Syndrome	G-button
P10	Female	Employee	Basic school	Male	17	Pulmonary hypertension	CVC O2 CN Nocturnal BiPAP

*Note*. BiPAP = bilevel positive airway pressure; CVC = central venous catheter; G-button = gastrostomy button; O2 CN = the nasal cannula; PEG = percutaneous endoscopic gastrostomy.

Two main themes were identified from the analysis of the data (Table 4) that described the essence of the lived experience of parents with technology-dependent children: (1) Discovering a new parenthood: parent and caregiver and (2) Reconciling daily life with the needs of the technology-dependent children. Each theme was divided into two subthemes; in the next section, we explore them in more detail.

**Table 4.** Themes, Subthemes, and Phenomenological Expressions.

Theme	Subtheme	Phenomenological expressions
1- Discovering a new parenthood: parent and caregiver	Family reorganization	Reorganization of family roles.
		The demands of disease.
	Learning to use a medical device	Complexity associated with new learning.
		Extra work and new responsibilities.
		Communication with healthcare professionals.
2- Reconciling daily life with the needs of the technology-dependent children	Importance of support systems	Informal support.
		Formal support.
		Coping strategies.
	Experiencing difficulties	Lack of external support
		Social inclusion and understanding.
		The uncertainty of the future concerns.

### Theme 1: Discovering a New Parenthood: Parent and Caregiver

The first theme contained findings about the profound transformation that parents go through when they become the main carers for a technology-dependent child. Parents described adjusting their daily routines to accommodate caregiving tasks and the process of adapting to new roles that included learning the skills needed to use their child’s medical device. The first experiences were described as shocking and ongoing emotional strain. Parents experienced initial difficulties in dealing with the child’s health problems and reported the need to adapt to complex care responsibilities gradually. Parents described that this dual role of being a parent and caregiver significantly changed the traditional understanding of parenting and described their experiences as complex and challenging. This theme included two subthemes: “Family reorganization” and “Learning to use medical device.”

#### Subtheme 1.1: Family Reorganization

The parents reported that the experience of caring for their child is different from having a healthy child, describing its multifaceted challenges. They reported that the complex and continuous care required and the time spent on it interfere with fulfilling other family and social responsibilities. For example, they are obliged to regularly attend treatments and/or hospital appointments and/or to keep a rigid schedule to care for their child. To fulfill their commitments, the parents described that family dynamics and routines were planned and carried out according to the child’s needs and health condition:

At first it was very scary, it was very complicated, and it was an adjustment. It’s not like having a healthy daughter. We have many more things to deal with, many more obligations. We have to deprive ourselves of our lives a little to fulfil her needs and give her a better standard of living. We no longer find it strange to have to come to the hospital ten times a month, for us coming here is a second home, it doesn’t make any difference to us coming here, more appointments, fewer appointments. I think it’s easier to cope now, but it’s still not 100 per cent. (P1)

Looking after my daughter isn’t very easy, because she takes medication, it always must be taken on time, she has therapies. It’s a bit complicated to do everything, but I must. My whole life has changed, completely 100 per cent. (P3)

Parents expressed that the level of care required, such as parenteral nutrition via a central catheter, needs constant supervision by a caregiver. Most parents reported that they share childcare tasks. However, mothers reported that they are the ones who care for the technology-dependent children, and fathers are the family support workers. In addition, when the child has siblings, the parents described that the siblings often collaborated with them in caring for the child:

You know, looking after my son takes hours, hours to connect the parenteral, to administer the medication, it takes hours. Sometimes it’s difficult, I’m not saying it isn’t. Fortunately, the brothers help, when the boy came home, I [mother] taught him and they help. Sometimes the [parenteral nutrition] machine beeps, for example, and they say: “Mum, I’ll go and check”, but they do everything right, they help a lot. Even with the material, they’re always very attentive: “oh mum, this is missing, oh mum, that’s missing”. (P5)

I [mother] became more and more to take care of her, and my husband went out more to work. (P9)

Other changes in family dynamics were mentioned by the parents, which include changes even in the couple dynamic itself due to the need to supervise the child:

My husband spends the night with him because I have more difficulty with noises (the oxygen ventilator), so I sleep in our room. (P10).

#### Subtheme 1.2: Learning to Use a Medical Device

At first, parents were shocked by the placement of the medical devices. All the parents felt fear and anxiety about the unknown, namely having to learn to manage the devices at home on their own. They described having to stay in the hospital to learn how to handle the devices, familiarize themselves with medical care, and feel confident about continuing care at home. This hospitalization period, according to the parents, ranged from two days to several weeks and was recognized as being of great importance but a difficult period in our lives:

The central catheter was very panicky for me. When I got to the hospital and the doctor said, “Your son is going home with a central catheter, he’ll have a machine with 24-hour medication”, I was terrified. (P10)

At the beginning, I was worried and distressed, I thought: “How am I going to manage a situation like this, looking after him at home?”. For me and my husband it was an obstacle. We were learning, and my son could not be discharged without us learning how to do it. Because there is a lot - there is the parenteral nutrition, the central catheter! I spent some time in the hospital with him to learn and gain experience. I was afraid I wouldn’t be able to do it, but we did it and we managed well. (P7)

Some parents also mentioned that they did not immediately realize that their child needed the device, while others mentioned that placing the device improved their child’s well-being:

In the beginning, we were not very familiar with it, and we thought that maybe it was not that necessary. Then we understood that the BiPAP was important and necessary. We insisted, and from then on, she [the daughter] ended up accepting it. Now, she often asks to put it on during the day because she feels it improves her breathing. Now with PEG, as she’s always saying she’s unwell, it’s challenging, because she doesn’t feel well and it always ends up causing us fear, because you don’t know whether you’re doing it right or wrong. (P4)

The parents described that the child’s health needs did not allow them to have a break, as comorbidities and complications often arise, requiring constant and even last-minute changes. They had to constantly adapt to new routines and manage their children’s changing needs, which included dealing with the emotional and practical aspects of their care. This inconstancy, according to them, carries a high psychological and social burden, especially as it is often only the parents who know how to deal with these situations. Some parents mentioned that they felt that healthcare professionals in the community lacked technical skills, for example, on how to handle devices for pediatric patients, which imposed a greater burden and preparation on parents to respond in critical and urgent situations:

When it’s a pressure alarm, we’re immediately worried, we open the equipment, we go to unclog the catheter and at any moment, it can block and we have to come to the hospital for the night. I always have to be prepared for that, the luggage ready [to stay in hospital]. I always have to be one step ahead (P6).

More than learning, it’s a question of adapting. There’s the initial shock and then we adjust, adapt and accept her health needs. Sometimes better, sometimes worse, but we’ve managed to cope with the challenges. (P4)

Parents identified and reflected on their experiences and the importance of effective communication with healthcare professionals to deal with different situations. According to them, the quality of communication significantly impacted the parents’ ability to manage their child’s condition and general well-being. Nurses played a key role in the experiences of our study participants. Thus, parents referred to nurses as a consistent source of support, emphasizing their attention, availability, and helpfulness:

At the time, the healthcare team that was here were tireless, they were always ready to listen to us, help, support, always very kind, for us it was essential. (. . .) The nurses were like another family to us. They adopted us and always showed concern and support, which made a huge difference. (P1)

We always had support from the doctors and nurses, we still keep in touch with Doctor X today. (. . .) I just have to say that they [nurses] are another family who didn’t know us from the side, and they adopted us, we always felt that support, concern, and it was very good for us.” (P10).

### Theme 2: Reconciling Daily Life with the Needs of the Technology-Dependent Children

After the initial impact and more experience in dealing with medical devices, parents begin to normalize their lives. This second theme explores the multifaceted support systems that parents rely on, including informal support from family and friends, and formal support. Although parents emphasized the importance of comprehensive support systems and strategies used to manage difficult circumstances and maintain hope for the future, they also described the difficult experiences they had to endure. This theme included two subthemes: “Importance of support systems” and “Experiencing difficulties.”

#### Subtheme 2.1: Importance of Support Systems

Family support is mentioned by parents as an asset to their well-being. Managing the social, financial, and emotional challenges of caring for a dependent technology child is burdensome, making the support provided at different levels immensely valuable. Parents emphasized that the unity, optimism, and collaboration of other family members in difficult times were strengths that helped them cope better with the whole situation. However, parents also reported that having a dependent technology child creates significant discomfort, fear, and apprehension among family members who are not directly involved in the daily care process. Consequently, these family members are often reluctant to take on direct care responsibilities. Instead, they prefer to assist with household chores, such as ironing and shopping. This type of support is most often provided by grandparents, especially grandmothers, who find these tasks easier to manage and less intimidating than the medical care itself.

I was always very supported by my parents. They live very close to us. My sister and sister-in-law too, they all supported me always. They tried to help me. Friends too. (P3).

My mother-in-law is a bit afraid. If it’s one or two hours she stays [with the child]. (P6).

The financial burden on parents due to frequent hospital visits and constant travel is significant. Many parents described the crucial role of family contributions, such as spontaneous donations of money or essential goods, which greatly alleviate this strain. However, not all families receive this support, either due to the absence of a close support network or a lack of awareness about available resources. This disparity has profound negative impacts, leaving some parents feeling isolated and overwhelmed.

“They [family] help monetarily for me to buy fuel. (. . .) In the first 3 months, all my colleagues came together and brought milk and diapers and food to the house to give my daughters” (P2).

The hardest thing was being alone. Without a close support network, all aspects of caring become more difficult. (P9)

Parents described formal support, which includes assistance from public and private organizations, as playing a crucial role in helping families overcome their challenges. One form of support frequently mentioned was the flexibility provided by employers, who authorized flexible working hours, which allowed parents to better coordinate family life and meet their children’s needs. Parents also recognize public and health services as an important support, as they provide expensive treatments free of charge, significantly reducing the financial burden on families. Other additional support, such as ambulance transport services for treatment and the presence and availability of volunteers to help during frequent hospitalizations, were also recognized:

My employer has been very understanding about my situation. They’ve allowed me to work from home when necessary and take time off for my child’s medical appointments. (P5)

Of the aids, financially it is the support of the Public State that co-pays the treatments, because that was inconceivable, that has a huge cost. (P8)

Despite these challenges, many parents described various coping strategies that help them adapt to the chronic condition. Throughout this process, families gained strength and fought to remain optimistic about their situation and their children’s future. One significant coping resource mentioned by parents was their faith and religion, which promoted their hope:

I rely on my faith to get through the toughest times. It gives me hope and strength to keep going. (P7)

Our life is as up and down like the beating of our heart because they say when it stops, it’s because we die. I try to face things like that, I’m lucky enough to be optimistic and face things with optimism and take it less seriously, to lighten the load a little bit. (P8).

Many parents and family members reported that they sought additional information and validation through online resources. While this helped them to understand the chronic condition better, it also induced anxiety due to misinformation and the negative portrayal of the disease. Some parents used these resources to keep abreast of research advances, although the bureaucracy involved in making treatments available was frustrating. Others found it helpful to liaise with families suffering from similar illnesses to share experiences and information:

The strategies I initially used were the Internet, but then I gave up, because there is a lot of misinformation. (. . .) At first, I followed on Twitter several scientific projects that are publishing new discoveries. But I realized, first they discover, they start testing, but from there until it becomes available it can take many years. So, I stopped the search because it was always increasing my anxiety. (P4).

I spoke to adults from Brazil, of whom there aren’t many here in Portugal, who I managed to speak to via the Internet and it was useful to share experiences and information. (P3).

#### Subtheme 2.2: Experiencing Difficulties

Contrary to what was expected, these families do not always have access to adequate resources and support networks, which exacerbates their difficulties. Some parents described a lack of external support to meet their informational, emotional, psychosocial, and practical care needs. For example, parents mentioned a lack of guidance regarding therapies and appropriate treatments, which increased feelings of helplessness. Additionally, there was a notable lack of information at the social resources level, with many parents unaware of their social rights. The absence of financial support also increased parental burden significantly. When mothers give up their jobs to provide full-time care, they often feel unprotected, as they lose financial support and social benefits.

The difficulties I had the most with [my daughter] was getting access to know what therapies she should do; I didn’t have guidance. (P3).

I have always worked, I have always paid my taxes and social security, and because I have a sick daughter, I could not continue working and I lost everything. (. . .) If I’m going to do something, I can’t do it because I don’t have a contract, so social security does not cover me. It like we don’t exist for society. (P9).

Parents described other social challenges, including a lack of understanding and empathy from the wider community. The visibility of their child’s condition often led to uncomfortable social interactions and families struggled with the lack of specialized services in schools:

When I go out in the street and he sometimes has those screaming crises, people are all staring. Then I must leave. People don’t understand! (P5).

At school she has no place to be. She needs to lie in a bed. The school is always complaining that there is no place for her to be there. (P9).

When a family has children, they idealize a future for their lives, but it can fall apart when faced with this experience. Given that some of the chronic conditions interfere with the children’s abilities to perform daily activities and/or regressions of their ability to perform them may arise, these parents are faced with constant uncertainty about the future of this child and their own life. This uncertainty brings suffering, anger, and fear to some of the parents, yet they express hope for the future:

I already knew beforehand that it was not going to be an easy recovery, and for now they have assured me that it is a success story, but I am always afraid of the future. (P6).

## Discussion

This study aimed to describe the lived experiences of parents of technology-dependent children. These parents, especially mothers, who are the main carers of these children in Portugal, must navigate a dual role that significantly alters their traditional understanding of parenting. This journey begins with an abrupt turnaround and a structural redefinition of family life and dynamics, due to the responsibility of caring for a child with complex and multifaceted health needs that are greater than those they had before or those of a healthy child.

Similar to other studies, our research found that families at home assume a variety of skills typically carried out by different professionals in a hospital setting (Toly, Blanchette, et al., 2019). The responsibilities include handling medical devices, medication management, adhering to strict treatment schedules, coordinating healthcare and education, managing transportation and appointment times with multidisciplinary teams. Additionally, parents must care for other family members and maintain other routine family responsibilities (Fisher et al., 2024; Mai et al., 2020).

In our study, like other research (Brenner et al., 2018; Toly, Blanchette, & Musil, 2019), parents often prioritize their children’s needs to the detriment of their own due to the relentless intensity of care provision. The cumulative impact of health challenges can be overwhelming for these families. This constant demand on their time and resources can jeopardize physical and emotional health as parents struggle to balance their caring responsibilities with other family and social obligations (Foster et al., 2022; Smith et al., 2022).

The journey of learning to use medical devices is a critical aspect of caregiving for parents of children with complex medical needs. These parents constantly experience feelings of fear, worry, and stress, both in relation to their children’s health and also in managing the medical device (Fernandez-Medina et al., 2022; Toly, Blanchette, et al., 2019). Parents reported the supportive role of health professionals in providing complete training, mostly in inpatient settings. This learning period is difficult but essential in helping parents overcome their fears and gain the confidence necessary to manage their children’s medical needs effectively. Empowerment through knowledge and skill promotes a sense of control and preparation, significantly improving the quality of life of both the child and the family (Amar-Dolan et al., 2020). However, despite feeling prepared for the routine care of their children, parents reported enormous pressure in situations that may be out of their control, such as emergencies. Always being one step ahead and organized for the possibility of having to rush to an emergency keeps these parents “on their toes.” Strategies such as training in simulation of critical and urgent situations can also help increase parents’ self-efficacy (Mai et al., 2020).

Integral to this journey of managing medical devices is the importance of effective communication with healthcare professionals. Parents identified and reflected on their experiences and the importance of effective communication with healthcare professionals to deal with different situations. According to them, the quality of communication, especially with nurses, had a significant impact on their overall well-being. Effective nurse-parent communication fosters trust and a sense of partnership, which is crucial for managing the multifaceted challenges of caring for technology-dependent children (Giambra et al., 2017). Nurses must be able to recognize the experience of these families and develop a relationship of trust, helping parents achieve a balance between their caregiving responsibilities and other roles (Toly, Blanchette, & Musil, 2019). Current nursing practices are increasingly focused on ensuring comprehensive care for the family unit. This includes not only managing medical devices and complex care for the child but also addressing the emotional and psychosocial needs of the entire family, promoting a holistic approach to care (Breneol et al., 2019; Shajani & Snell, 2023).

The participants reported that both formal and informal support systems were crucial to managing the burdens associated with caring for their children. Informal support, whether from close relatives or friends, provided emotional and instrumental support, which was fundamental to their well-being and the fulfillment of their daily responsibilities. Formal support, including from their employers and public and private services, was also considered vital. The lack of this support network can worsen the challenges of providing care and increase the tensions and challenges faced in everyday life, making it difficult to maintain the physical and mental health of parents, as found in this study and also evidenced by the literature (Chan et al., 2019; Yang et al., 2022).

Parents of technology-dependent children often face social stigma and a lack of understanding from the wider community, which can manifest itself in uncomfortable social interactions and a notable lack of empathy for the unique challenges these families face. This social attitude exacerbates the stress and isolation experienced by these families, making their roles as carers even more challenging (Chan et al., 2019). In addition, many parents report a lack of specialized services in schools, which further complicates their efforts to provide adequate care and support for their children. Schools often lack the necessary resources and trained staff to accommodate the unique needs of technology-dependent children, leading to inadequate educational experiences and increased pressure on families (Toly, Blanchette, & Musil, 2019).

Understanding what support is needed and having assessment strategies to identify the needs of each family is important for improving the multidisciplinary interventions of the professionals who work in collaboration with the family (Giambra & Spratling, 2023). As highlighted by Thomas et al. (2023), investing in specialist training to support parents caring for children with medical devices at home is essential. Furthermore, developing comprehensive support programs should be a societal priority to reduce inequalities. These programs could also include financial counselors to help families access resources and financial assistance programs to alleviate financial stress (Giambra & Spratling, 2023).

Despite these challenges, most parents acquire the necessary skills and knowledge to manage the chronic condition with a positive attitude, enabling them to deal effectively and efficiently with the difficulties experienced. In challenging situations, they are able to draw on their hope and faith, maintaining an optimistic vision for their future. This resilience and adaptability are highlighted in the literature (Huang et al., 2022; Toly, Blanchette, et al., 2019; Tsibidaki, 2020).

Although many studies have explored the experiences of parents with technology-dependent children in various countries, our study provides a unique focus on the Portuguese context. This research has highlighted the challenges these parents face and the support they need, identifying unique contextual factors and commonalities that can serve as a basis for planning better healthcare practices and policies in the Portuguese context. Furthermore, understanding these unique contextual factors can provide useful information for developing more effective support strategies in Portugal and similar contexts.

### Strengths and Limitations

Our study provides a unique and in-depth exploration of the lived experiences of parents with technology-dependent children, a topic that has been under-researched in Portugal. However, there are limitations that should be highlighted. Firstly, the sample only included Portuguese-speaking parents (from Portugal and Brazil) who came from a single geographical area of a single country. Similar studies should be carried out in other geographic settings. Secondly, the parents’ characteristics are similar, which does not guarantee variability of responses. In addition, most of the participants were women. Future research should include the perspective of fathers, unmarried and/or cohabiting parents, other family members, and healthcare professionals to gain a deeper and more comprehensive understanding of the topic.

### Implications for Future Practice

This study provides insights for healthcare professionals and stakeholders to develop interventions that support these families, responding to their healthcare, emotional, and social needs. Focus should be directed toward investing in the training of health professionals working in the community, namely in the acquisition of advanced pediatric skills and an in-depth understanding of the unique challenges faced by these families. In this way, the quality of care and support can be significantly improved.

The development of action algorithms as tools to support standardized communication and the coordination of care can guarantee coherent, clear, and empathetic communication on the part of healthcare professionals and improve coordination and collaboration between multidisciplinary teams. These algorithms also allow parents to receive consistent, comprehensive, and compassionate support throughout their journey, which can significantly alleviate their concerns and improve their ability to provide care. The creation of multidisciplinary support teams that include health professionals and social workers can provide holistic support, responding to families’ medical, emotional, and financial needs.

Also, home health nursing and the development of respite care programs can provide much-needed breaks for primary caregivers, helping to alleviate caregiver burnout and maintain their well-being. In addition, the development of new support policies and the increase in campaigns to raise awareness in society about the challenges these families face is essential to create a more supportive and inclusive community.

## Conclusion

While many studies have explored the experiences of parents with technology-dependent children in various countries, our study provides a unique focus on the Portuguese context. This geographic specificity highlights distinct cultural, social, and healthcare system-related factors that influence the caregiving experience. In this study, the whole transformation in the lives of these families leads them to make considerable changes in their family roles and daily routines, particularly because of the need for complex and constant health care for the child. They experience high levels of stress and anxiety, which start from the moment the need for a device is communicated and continue during the process of learning and providing care, in addition to changes in family dynamics.

Parents reported that managing the social, financial, and emotional challenges of caring for a technology-dependent child is a heavy burden. Despite these challenges, having an informal support network of family and friends, as well as formal support, helps them cope with their demanding responsibilities. This new and deeper understanding of the experiences of Portuguese parents provides a basis for nurses and other healthcare professionals to establish a more family-centered healthcare system and improve future nursing practices. We hope that, based on the results of this study, new strategies can be developed to offer better support to these families.
